# The circular RNA circMAST1 promotes hepatocellular carcinoma cell proliferation and migration by sponging miR-1299 and regulating CTNND1 expression

**DOI:** 10.1038/s41419-020-2532-y

**Published:** 2020-05-11

**Authors:** Xiufeng Yu, Ping Sheng, Jing Sun, Xijuang Zhao, Junting Zhang, Yiying Li, YiMeng Zhang, Wenxiu Zhang, Jianqi Wang, Kunpeng Liu, Daling Zhu, Hongchi Jiang

**Affiliations:** 1 Department of Hepatic Surgery, The First Affiliated Hospital of Harbin Medical University, Key Laboratory of Hepatosplenic Surgery, Ministry of Education, Harbin, 150081 China; 2 College of Medical Laboratory Science and Technology, Harbin Medical University (Daqing), Daqing, 163319 China; 3 Central Laboratory of Harbin Medical University (Daqing), Daqing, 163319 China; 4 College of Bioinformatics and Technology, Harbin Medical University (Daqing), Daqing, 163319 China

**Keywords:** Oncogenes, Small RNAs

## Abstract

Circular RNAs (circRNAs) are a class of non-coding RNAs with a loop structure; however, their functions remain largely unknown. Growing evidence suggests that circRNAs play a pivotal role in the progression of malignant diseases. However, the expression profiles and function of circRNAs in hepatocellular carcinoma (HCC) remain unclear. We investigated the expression of microtubule-associated serine/threonine kinase 1 (MAST1) circRNA (circMAST1) in HCC and healthy tissues using bioinformatics, quantitative real-time PCR (qRT-PCR), and fluorescence in situ hybridization. Luciferase reporter assays were performed to assess the interaction between circMAST1 and miR-1299. Proliferation assays, colony formation assays, flow cytometry, transwell assays, and western blotting were also performed. A mouse xenograft model was also used to determine the effect of circMAST1 on HCC growth in vivo. CircMAST1 was upregulated in HCC tissues and cell lines; silencing via small interfering RNA inhibited migration, invasion, and proliferation of HCC cell lines in vitro as well as tumor growth in vivo. Furthermore, the expression of circMAST1 was positively correlated with catenin delta-1 (CTNND1) and negatively correlated with microRNA (miR)-1299 in HCC clinical samples. Importantly, circMAST1 sponged miR-1299 to stabilize the expression of CTNND1 and promoted tumorigenic features in HCC cell lines. We found that circMAST1 may serve as a novel biomarker for HCC. Moreover, circMAST1 elicits HCC progression by sponging miRNA-1299 and stabilizing CTNND1. Our data provide potential options for therapeutic targets in patients with HCC.

## Introduction

Hepatocellular carcinoma (HCC) is one of the most common malignant tumors globally and constitutes the third leading cause of cancer-related deaths worldwide^1^. In 2017, there were 953,000 cases of liver cancer and 819,000 deaths globally^2^. HCC is clinically characterized by its invasiveness, poor prognosis, and limited therapeutic options. Despite the advances in the clinical understanding of the mechanisms of HCC, the 5-year survival rate of patients with this disease remains low^3^. Presently, surgery is the most common intervention for patients with HCC, but most patients with multifocal development and distant metastases are ineligible for curative surgical treatment^4^. Therefore, a deeper understanding of the molecular mechanisms underlying HCC progression is of paramount importance.

Circular RNAs (circRNAs) are a newly discovered type of non-coding RNAs^5,6^. Unlike canonical linear RNAs, circRNAs are highly conserved and are characterized by covalently linked closed-loop structures with neither 5′-3′ caps nor polarity; they also have no polyadenylated tails, which makes them more stable than linear RNAs^7^. The biological function of circRNAs has been extensively investigated and can be grouped into five categories; sponging of microRNAs (miRNAs) to suppress their function^8^, transcriptional and translational regulation^9,10^, influencing alternative splicing of pre-mRNAs^11,12^, interacting with RNA-binding proteins to regulate gene expression^13^, and potentially, encoding proteins^14,15^. These features collectively indicate pivotal functions for circRNAs in biological and pathological processes.

A growing body of literature shows that circRNAs are differentially expressed in HCC^16–19^. Some studies have demonstrated that circRNAs act as oncogenes in HCC^20–22^, while others showed that they act as HCC tumor suppressors^18,19,23–25^. As such, circRNAs are implicated in the onset and development of HCC. CircRNAs are also highly abundant and stable, suggesting that they are ideal biomarkers and promising therapeutic targets for patients with HCC. However, compared with other non-coding RNAs such as miRNAs and long non-coding RNAs, the study of circRNAs in HCC is just beginning. To date, only a small quantity of functional circRNAs have been discovered and characterized in HCC^26^; a large number remain to be explored or identified.

In this study, we aimed to find out if circMAST1 can serve as a biomarker as well as a potential therapeutic target for patients with HCC, by analyzing the expression profile of circRNAs in HCC tissues. We determined that the circRNA of microtubule-associated serine/threonine kinase 1 (MAST1), or circMAST1, was significantly upregulated in HCC tissues and was closely related to tumor progression through a novel route.

## Results

### Profiles of circRNAs in HCC

**Fig. 1:**
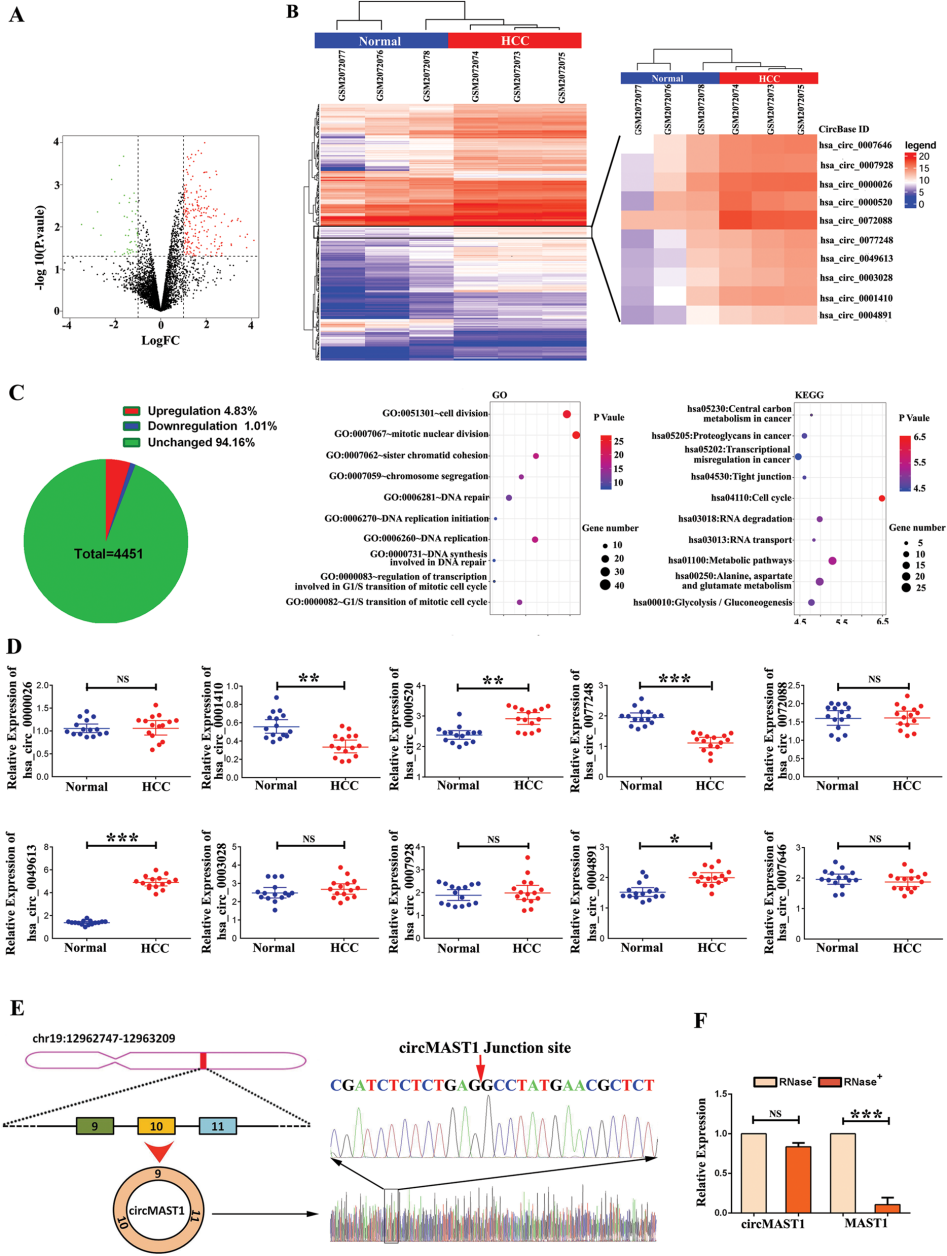
CircRNA expression profiles in HCC and paired non-tumorous samples. **a** The volcano plot was constructed with fold-change values and *p*-values. The horizontal line corresponds to a *p*-value of 0.05, and the vertical lines indicate upregulation and down-regulation by 2-fold. The red and green points on the volcano plot indicate the differentially expressed circRNAs with a greater than 2-fold change between the two compared groups. **b** Heat map depicts the differentially expressed circRNAs with a greater than 2-fold in 3 paired human HCC tissues and matched normal tissues. Red represents up-regulated circRNAs, and blue represents down-regulated circRNAs. **c** KEGG and GO pathway analysis of the parental genes of differertially expressed circRNAs (Hypergeometric test). the top of ten categories are exhibited. **d** The relative expression of ten most upregulated circRNAs (hsa_circ_0000026, hsa_circ_0001410, hsa_circ_0000520, hsa_circ_0077248, hsa_circ_0072088 hsa_circ_0049613, hsa_circ_0003028, hsa_circ_0007928, hsa_circ_0004981, hsa_circ_0007646)(*P < 0.05; **P < 0.01; ***P < 0.001; *n* = 15). **e** Schematic illustration showed that circMAST1 is located at chromosome 19p13.2 and cyclized from exons 9–11 of MAST1, the PCR products of circMAST1 were confirmed by Sanger sequencing. **f** qRT-PCR analysis of circMAST1 and MAST1 mRNA after treatment with or without RNase R in HCC tissues (***P < 0.001; *n* = 4). HCC, hepatocellular carcinoma; qRT-PCR, quantitative reverse transcription polymerase chain reaction.

A total of 4451 circRNAs were detected in three pairs of HCC tissue samples (3 HCC tissues and three matching non-tumor liver tissues) using circRNA microarray analysis from the microarray dataset GSE78520. Using the “limma” package of the R software and selecting a false discovery rate of less than 0.05 and |log2fold-change| greater than 1 as cut-off criteria, we identified 257 differentially expressed circRNAs, of which 213 and 44 were upregulated and downregulated, respectively (Additional file 6: Supplementary Table S5). Volcano and scatter plots showed the variation in circRNA expression between the HCC tissues and non-tumor liver tissues (Fig. 1a). A heatmap representing the differentially expressed circRNAs values was generated to distinguish human liver cancer from adjacent healthy liver tissues (Fig. 1b). Afterwards, the parental genes of differentially expressed circRNAs were subjected to ‘Kyoto Encyclopedia of Genes and Genomes (KEGG)’ pathway analysis; the top ten entries are shown in Fig. 1c. These parental genes regulate cell division, mitotic nuclear division, sister chromatid cohesion, chromosome segregation, DNA repair, replication initiation, DNA replication, DNA synthesis involved in DNA repair, regulation of transcription involved in the G1/S transition of the mitotic cell cycle, and G1/S transition itself.

Next, we detected ten circRNAs expressed in both HCC tissues and paired non-tumor tissues from 15 patients using qRT-PCR. Among them, we found that hsa_circRNA_102459 (hsa_circ_0049613, circMAST1) expression was persistently and significantly increased in HCC compared to that of the matching adjacent normal liver tissues (Fig. 1d). We also detected the existence of circMAST1 in the serum of HCC patients and healthy controls. The results indicated that the serum levels of circMAST1 were significantly higher in patients with HCC than that of healthy controls (Additional file 7: Supplementary Fig. S1A). Thus, we focused on investigating the role of circMAST1 in HCC progression. Notably, circMAST1 was derived from exons 9–11 of *MAST1* located on chromosome 19p13.2 and independent experiments were performed to determine its circular structure. We first inserted the PCR products of circMAST1 into a T vector for Sanger sequencing (Fig. 1e), which showed consistency with the back spliced region of circMAST1 supplied by circBase^27^. The circular structure of circMAST1 was confirmed using RNase R. As shown in Fig. 1f, the linear and circular transcripts of MAST1 were amplified in HCC tissues; the linear transcripts of MAST1 were degraded by RNase R, while the circular transcripts of MAST1 were resistant to degradation. The data demonstrate both the presence and circular structure of circMAST1.

### circMAST1 is mainly located in the cell cytoplasm

**Fig. 2:**
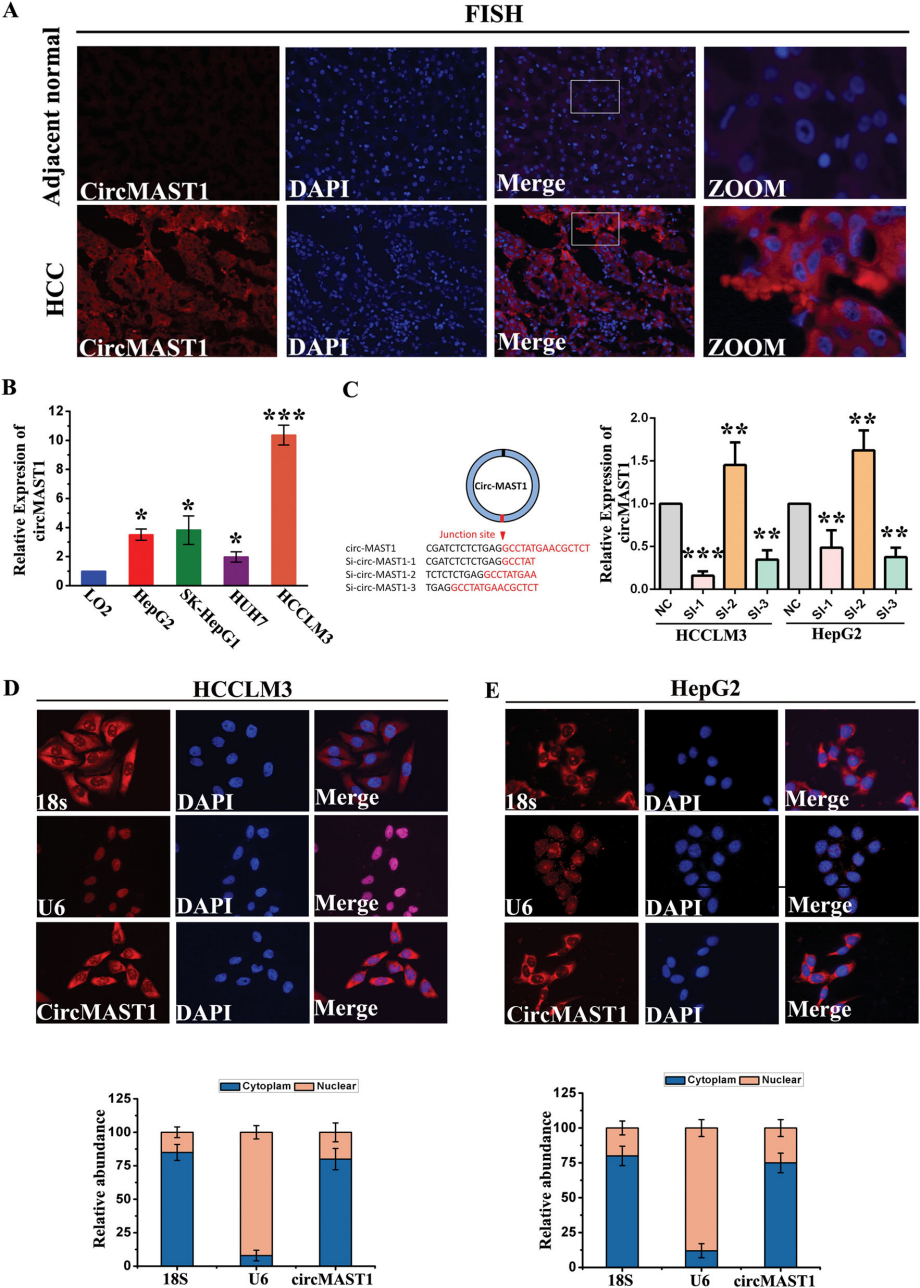
circMAST1 locates in cytoplasm. **a** circMAST1 in HCC adjacent non-tumor and tumor tissues was detected by FISH. **b** The expression levels of circMAST1 in multiple HCC cell lines (*P < 0.05; ***P < 0.001; *n* = 4). **c** Illustration showing the siRNA targeting the back-splice junction (si-circMAST-1, si-circMAST1-2 and si-circMAST1-3); qRT-PCR results for circMAST1 in HCLLM3 and hepG2 cells treated with or without siRNA (si-NC, control oligonucleotides with scramble sequence; si-circMAST-1, si-circMAST1-2 and si-circMAST1-3, oligonucleotides targeting the back-splice junction) (**P < 0.01; ***P < 0.001; *n* = 4). **d**, **e** FISH confirmed that circMAST1 was predominantly located in cytoplasm. Nuclei were stained with DAPI. u6, 18s and circMAST1 were labeled with cy3.

In general, the subcellular localization of circRNA determines its primary mode of action. The FISH analysis revealed that circMAST1 level was higher in tumor tissues than in the matching non-tumor counterparts (Fig. 2a). Moreover, comprehensive assessments of circMAST1 expression in the HCC cell lines HepG2, SK-HepG1, Huh7, and HCCLM3, as well as in healthy liver L02 cells, were performed using qRT-PCR. The expression levels of circMAST1 in all HCC cell lines were generally higher than that of L02 cells, the highest observed in HCCLM3 cells and lowest in Huh7 cells (Fig. 2b). To further investigate the regulatory role of circMAST1, we designed three circMAST1 small interfering RNAs (siRNAs) to specifically target different binding sites on the back splice junction sequence of circMAST1; in both the HCCLM3 and HepG2 cell lines, siRNA-1 and siRNA-3 effectively silenced the expression of circMAST1 and were used for subsequent experiments (Fig. 2c). Moreover, circMAST1 was predominantly located in the cytoplasm as confirmed by FISH (Fig. 2d, e). The results indicate that circMAST1 is a highly stable cytoplasmic circRNA derived from exons 9–11 of the *MAST1* locus.

### circMAST1 is likely required to sustain HCC cell growth in vitro

**Fig. 3:**
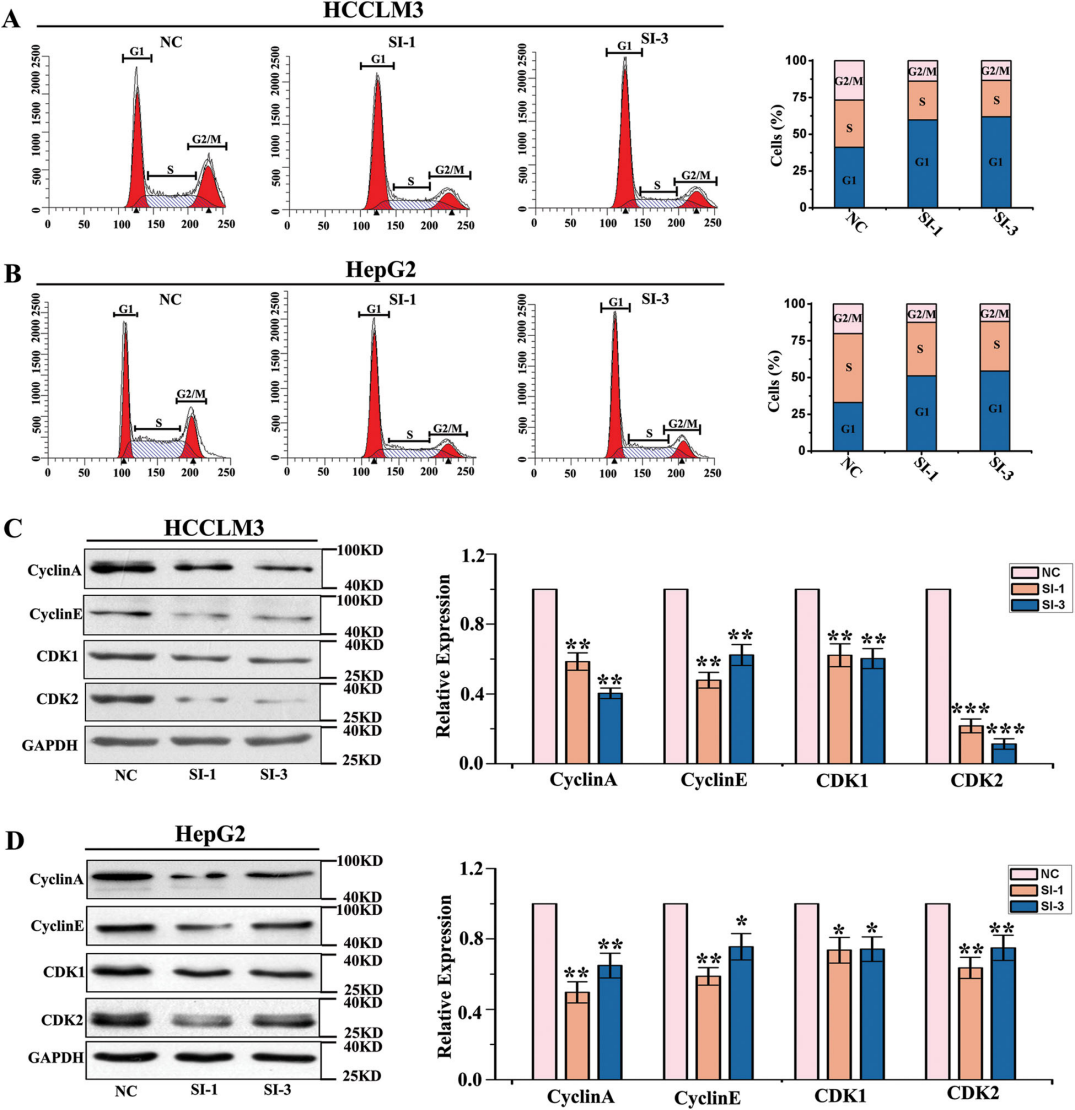
circMAST1 affects HCC cell cycle. **a**, **b** The number of cells in each phase of the cell cycle was examined by FACS analysis. Bar graph showed the results of the number of cells in each phase of the cell cycle (*n* = 3). **c**, **d** The expression of cyclin A, cyclin E and CDK1, CDK2 in HCLLM3 and hepG2 cells by Western-blot. Bar graph showed the results of the expression of cyclin A, cyclin D and CDK1, CDK2 in HCLLM3 and hepG2 cells (*P < 0.05; **P < 0.01; ***P < 0.001; *n* = 4). NC, control oligonucleotides with scramble sequence; SI-1, si-circMAST-1;SI-3, si-circMAST1-3.

**Fig. 4:**
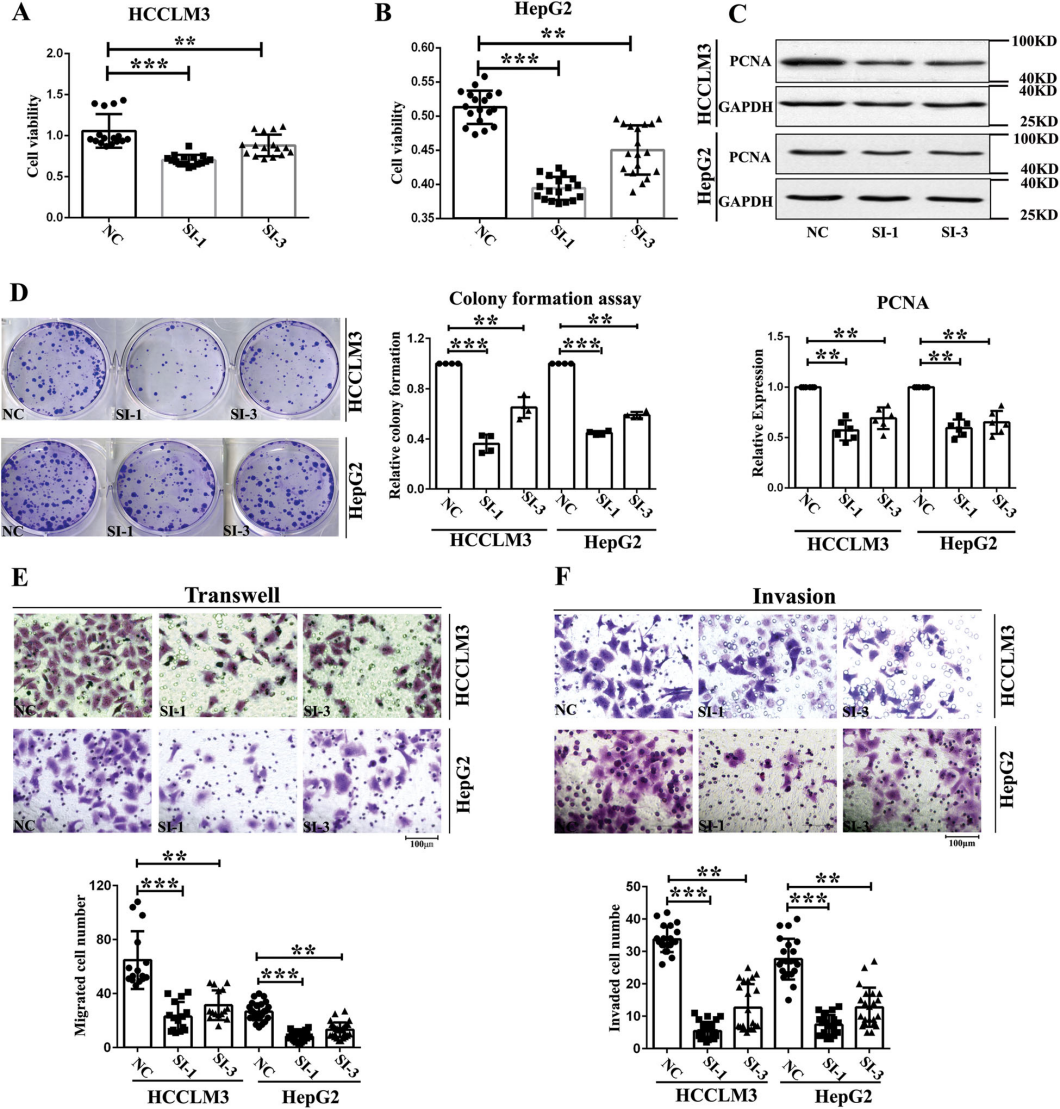
circMAST1 promotes the proliferation, migration, and invasion of HCC cells in vitro. **a**, **b** Cell proliferation ability of HCLLM3 and HepG2 cells transfected with siRNA-circMAST1 or siRNA-NC was evaluated by WST-1 assay (**P < 0.01; ***P < 0.001; *n* = 4). **C**. PCNA protein expression was determined after knockdown of siRNA-circMAST1 or siRNA-NC in HCLLM3 and HepG2 (**P < 0.01; *n* = 6). **d** Colony formation assay was used evaluated the proliferation ability of HCLLM3 and hepG2 cells transfected with siRNA-circMAST1 or siRNA-NC (**P < 0.01; ***P < 0.001; *n* = 4). **e** Cell migration capability of HCLLM3 and hepG2 cells transfected with siRNA-circMAST1 or siRNA-NC was assessed by transwell migration (**P < 0.01; ***P < 0.001; *n* = 4). **f** Cell invasion capability of cells transfected with siRNA-circMAST1 or siRNA-NC was evaluated by matrigel nivasion assays(**P < 0.01; ***P < 0.001; *n* = 4). NC, control oligonucleotides with scramble sequence; SI-1, si-circMAST-1;SI-3, si-circMAST1-3.

Using the back splice junction-specific siRNA, we successfully silenced circMAST1 expression in HCCLM3 and HepG2 cells. We then investigated whether circMAST1 affected HCC cell cycle progression using flow cytometry. Silencing circMAST1 increased the proportions of HCCLM3 and HepG2 cells in G0/G1 phase significantly while decreasing the proportions of these cells in the G2 phase (Fig. 3a, b). Furthermore, circMAST1 was found to strongly affect the post-translational levels of the cell cycle-related proteins cyclin A and cyclin E as well as the cyclin-dependent kinases (CDKs) 1 and 2 in these cells (Fig. 3c, d). The WST-1 assay showed that circMAST1 silencing reduced HCCLM3 and HepG2 cell proliferation (Fig. 4a, b). Notably, proliferating cell nuclear antigen (PCNA) levels in these cells were initially elevated but attenuated after transfection with the circMAST1 back splice junction-specific siRNA (Fig. 4c). Meanwhile, colony formation assays revealed that circMAST1 was positively associated with the proliferation of HCCLM3 and HepG2 cells (Fig. 4d). Moreover, HCCLM3 and HepG2 cell migration were suppressed by circMAST1 silencing (Fig. 4e), as was the cell invasion through the Matrigel (Fig. 4f). We generated Huh-7 cells stably overexpressing circMAST1 by transducing them with the lentivirus of circMAST1. In comparison with the controls, circMAST1 overexpression increased the proliferation, migration, and invasion of Huh-7 cells (Fig S2A-C). These results suggest that circMAST1 is likely required to sustain the proliferation, migration, and invasion of HCC cells in vitro.

### circMAST1 is likely required to sustain HCC tumor growth in vivo

**Fig. 5:**
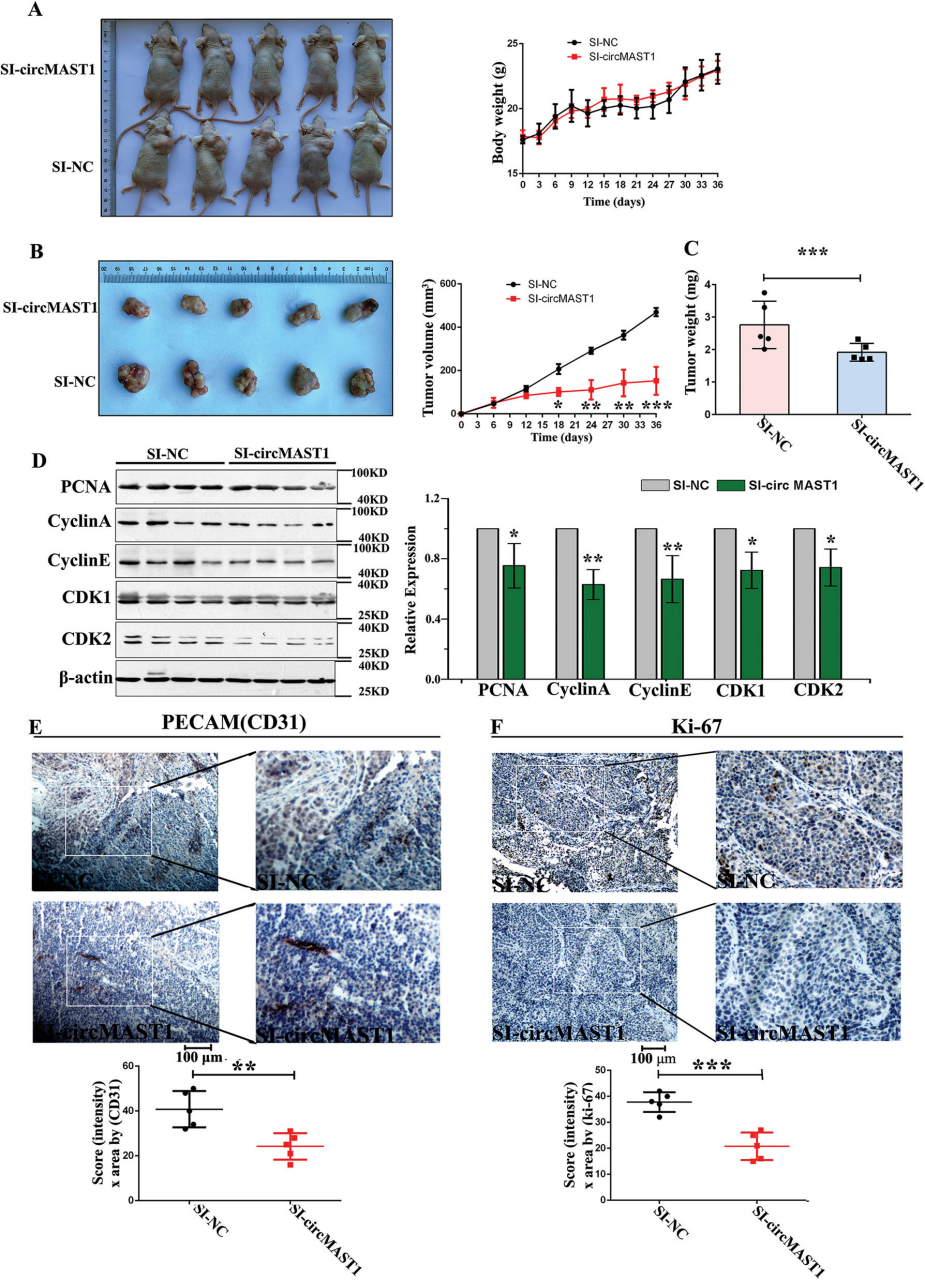
circMAST1 promotes HCC tumor growth in vivo. **a** Representative images of the HCC tumor bearing BALB/c nude mice. The body weight of x-enograft nude mice. **b** The tumor volumes were measured every 3 days (*P < 0.05; **P < 0.01; ***P < 0.001; *n* = 5). **c** The relative weights of tumors were evaluated (***P < 0.001; *n* = 5). **d** The expression of PCNA, cyclin A, cyclin E and CDK1, CDK2 in HCLLM3 and hepG2 cells by Western-blot. Bar graph showed the results of the expression of PCNA, cyclin A, cyclin D and CDK1, CDK2 in live tissue from tumor xenografts(*P < 0.05; **P < 0.01; *n* = 4). **e**, **f** IHC staining and IHC analysis of CD31 and ki67 expression in subcutaneous x-enograft tumors. Knockdown of circMAST1 could down-regulate CD31 and ki67 expression (*P < 0.05; **P < 0.01; ***P < 0.001; *n* = 5).

HCCLM3 cells were injected subcutaneously into BALB/c nude mice for 12 days to create tumor xenografts to examine the role of circMAST1 in HCC tumorigenesis further. Twelve days later, we commenced the injection of 10 OD cholesterol-modified circMAST1 siRNA or negative control siRNA every three days subcutaneously at the tumor site for 24 days (Fig. 5a). Following 36 days of observation, the mice that had received circMAST1 siRNA exhibited markedly decreased tumor volumes and weights than those that had received control siRNA (Fig. 5b, c), suggesting that circMAST1 promoted HCC cell growth in vivo. These results further support a role for circMAST1 in HCC tumorigenesis and development. At the same time, we examined the expression of PCNA protein as well as that of the cell cycle-related proteins cyclin A, cyclin E, CDK1, and CDK2 in the tumor tissues of circMAST1 siRNA- and control siRNA-injected mice; all were significantly lower in the circMAST1 siRNA group than the control siRNA group (Fig. 5d). Moreover, circMAST1 silencing significantly reduced the Ki-67 proliferation index as well as the number of CD31-positive intratumoral microvessels (Fig. 5e, f). These results verify that circMAST1 is likely required to sustain HCC growth in vivo.

### circMAST1 serves as a sponge for miR-1299 in HCC cells

**Fig. 6:**
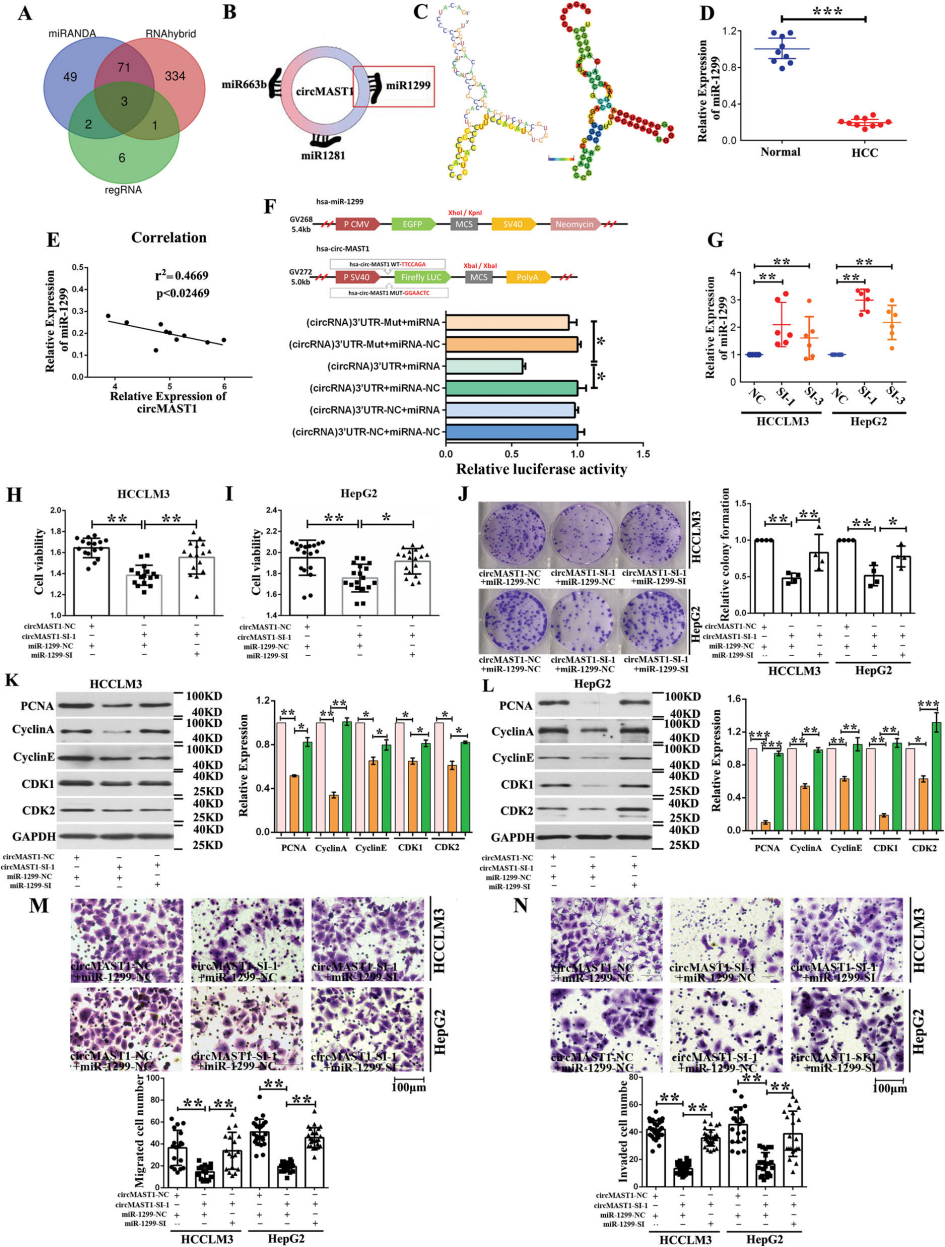
circMAST1 modulates the proliferation, cell cycle, migration, and invasion of HCC cells via miR-1299. **a** Three potential target miRNAs of circMAST1 were predicted by miRANDA, RNAhybrid and regRNA. **b** Schematic drawing showing the miRNAs that might bind circMAST1. **c** The possible binding sites of the 5’UTR of G3BP2 with miR-1299 were predicted by RegRNA2.0. Graph of predicted RNA secondary structure. The yellow region indicates the RNA-fold predicted structure of the motif. RNAfold reliability information of pair probabilities. Minimum free energy = −34.02. **d** Relative Expression of miR-1299 in HCC and normal tissue pairs were measured by qRT-PCR (***P < 0.001; *n* = 10). **e** Pearson’s correlation analyses showing the correlation of circMAST1 and miR-1299 expression (*n* = 10). **f** Schematic of circMAST1 wild type(wt) and mutant(mut) luciferase reporter vectors. luciferase reporter assay in HEK293T cells co-transfected with miRNA mimics, circMAST1 wild type (circMAST1-wt) and mutant (mut) luciferase reporter vectors. **g** The expression of miR-1299 were analyzed by using qRT-PCR in cells transfected with siRNA-circMAST1-1, siRNA-circMAST1-3 or siRNA-NC (**P < 0.01; *n* = 6). **h**, **i** The viability of HCLLM3 and hepG2 cells were measured after co-transfected siRNA-circMAST1 and miR-1299 inhibitor in by using WST-1 assays (*P < 0.05; **P < 0.01; *n* = 4). **j** Cell proliferation ability of HCLLM3 and hepG2 cells co-transfected with siRNA-circMAST1-1 and miR-1299 inhibitors were evaluated by colony formation assay (*P < 0.05; **P < 0.01; *n* = 4). **k, l** The expression of PCNA, cyclin A, cyclin E and CDK1, CDK2 in HCLLM3 and hepG2 cells by Western-blot. Bar graph showed the results of the expression of PCNA, cyclin A, cyclin D and CDK1, CDK2 in HCLLM3 and hepG2 cells (*P < 0.05; **P < 0.01; ***P < 0.001; *n* = 4). **m**, **n** Cell migration or invasion assays were performed in HCLLM3 and hepG2 cells co-transfected with siRNA-circMAST1-1 and miR-1299 inhibitors by using transwell chamber with or without matrigel respectively (**P < 0.01; *n* = 4).

Since circRNAs that are predominantly located in the cytoplasm are usually associated with miRNA sponging, we further explored whether circMAST1 could bind to miRNAs (Fig. 6a). Three miRNAs associated with circMAST1 (miR-663b, miR-1281, and miR-1299) were predicted to be relevant by miRanda, RNAhybrid, and regRNA; miR-1299 was selected as a candidate miRNA for subsequent experiments (Fig. 6b). Figure 6c illustrates the predicted RNA secondary structure; the yellow region indicates the predicted ‘RNAfold’ structure of the motif using calculated pair probabilities; the minimum free energy = −34.02. Previous studies showed that miR-1299 is downregulated in HCC tissues and functions as a tumor suppressor and that the low expression of miR-1299 in HCC tissues predicts poor prognosis (Fig. 6d). Pearson’s correlation analysis of circMAST1 and miR-1299 expression levels revealed a negative correlation (Fig. 6e). Additionally, luciferase reporters using either a wildtype circMAST1 sequence or a sequence with mutated miR-1299 binding sites, which were inserted into the 3′ untranslated region of Renilla luciferase, showed that miR-1299 overexpression significantly reduced the luciferase activities of the wildtype reporter but not the mutant sequence-bound reporter (Fig. 6f). Furthermore, circMAST1 silencing increased miR-1299 levels in HepG2 and HCCLM3 cells, as shown using qRT-PCR (Fig. 6g). These findings indicated that circMAST1 acts as a sponge for miR-1299.

### circMAST1 modulates the proliferation, cell cycle, migration, and invasion of HCC cells via miR-1299

For further investigation of whether circMAST1 affects the function of HCC cells via miR-1299, we co-transfected siRNA-circMAST1 and a miR-1299 inhibitor into HepG2 and HCCLM3 cells to determine whether circMAST1 exerts its tumor-promoting effect by sponging miR-1299. We found that the proliferation (via a WST-1 assay) and colony-forming ability of HCC cells co-transfected with siRNA-circMAST1 and miR-1299 inhibitor were higher than those of HCC cells transfected only with siRNA-circMAST1, suggesting that the downregulation of miR-1299 could partially reverse the inhibitory effect siRNA-circMAST1 had on proliferation (Fig. 6h–j). Similarly, western blots showed that levels of PCNA protein, cyclins A and E, and CDK1/2 were partly decreased in HCC cells co-transfected with siRNA-circMAST1+miR-1299 inhibitor (Fig. 6k, l). Meanwhile, the decrease in HepG2 and HCCLM3 migration and invasion caused by circMAST1 silencing could be reversed with miR-1299 inhibition (Fig. 6m, n). Collectively, these results demonstrated that circMAST1 is required to sustain HCC cell progression partly by impairing the tumor suppressor miR-1299.

### miR-1299 targets CTNND1, which is required to sustain HCC cell tumorigenicity

**Fig. 7:**
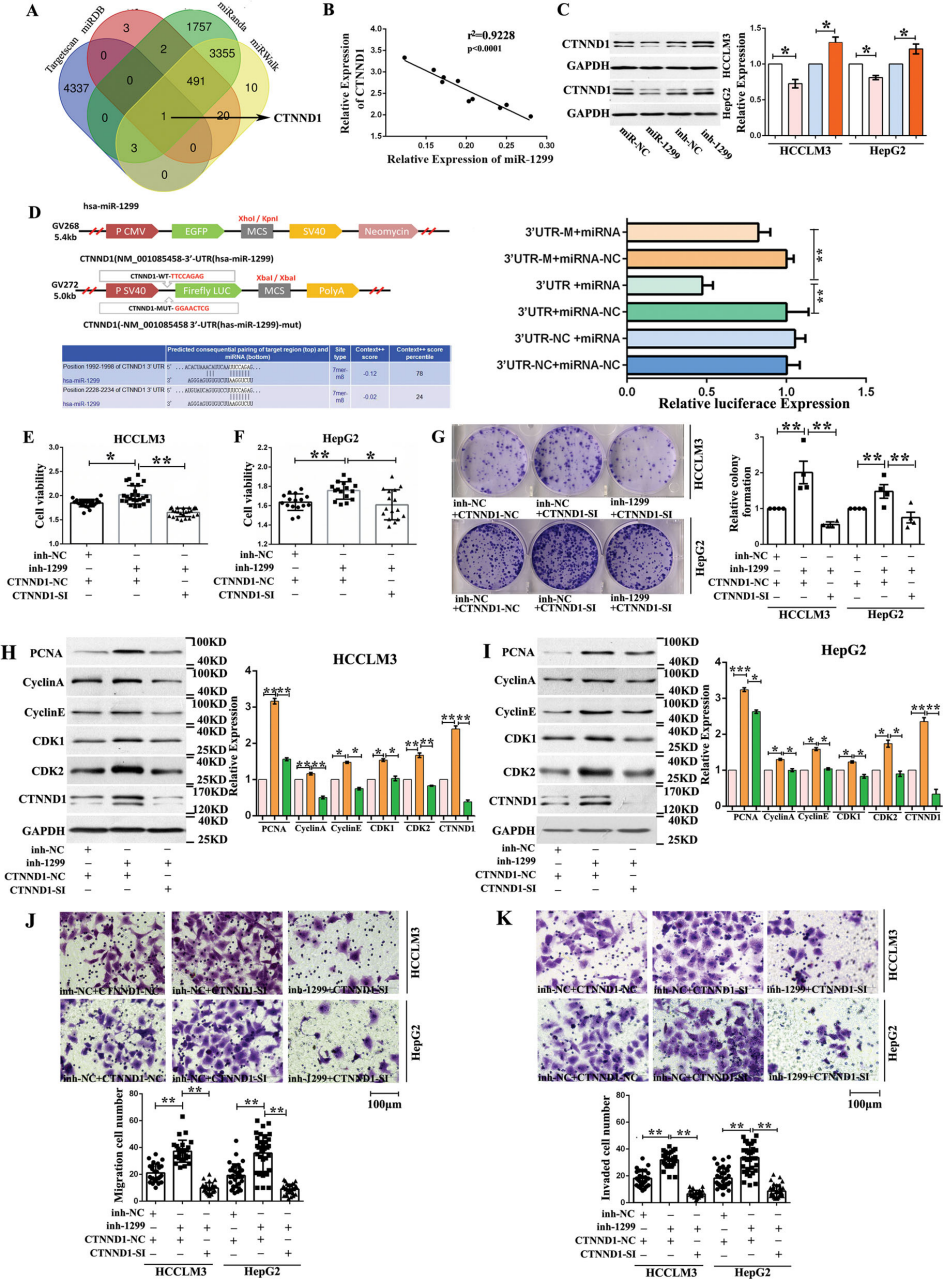
miR-1299 targets CTNND1 to sustain HCC cell tumorigenicity. **a** Venn diagram showing 1 genes that are putative miR-1299 targets computationally predicted by four algorithms (miRanda, miRDB, miRWalk and TargetScan). **b** Pearson’s correlation analyses showing the correlation of miR-1299 and CTNND1 expression (*n* = 10). **c** Western-blot analysis indicated that miR-1299 could down-regulate CTNND1 (*P < 0.05; *n* = 4). **d** Schematic of CTNND1 3’UTR wild-type (WT) and mutant (Mut) luciferase reporter vectors is shown. The relative luciferase activities were analyzed in 293T cells cotransfected with miR-1299 mimics or miR-NC and luciferase reporter vectors CTNND1 3’UTR (WT) or CTNND1 3’UTR (Mut) (**P < 0.01; *n* = 3). **e**, **f** The viability of HCLLM3 and hepG2 cells were measured after co-transfected with siRNA-CTNND1 and miR-1299 inhibitor in by using WST-1 assays (*P < 0.05; **P < 0.01; *n* = 4). **g** Cell proliferation ability of HCLLM3 and hepG2 cells co-transfected with siRNA-CTNND1 and miR-1299 inhibitor were evaluated by colony formation assay (**P < 0.01; *n* = 4). **h**, **i** The expression of PCNA, cyclin A, cyclin E and CDK1, CDK2, CTNND1 in HCLLM3 and hepG2 cells by Western-blot. Bar graph showed the results of the expression of PCNA, cyclin A, cyclin D and CDK1, CDK2, CTNND1 in HCLLM3 and hepG2 cells (*P < 0.05; **P < 0.01; *n* = 4). **j**, **k** The influence on cell migration and invasion abilities of HCLLM3 and hepG2 cells transfected with siRNA-CTNND1 and miR-1299 inhibitor was assessed by transwell migration and matrigel invasion assays (**P < 0.01; *n* = 4).

The miRanda, miRDB, TargetScan, and miRWalk prediction tools revealed high scores for miR-1299 targeting the 3′ untranslated region of the CTNND1 (Fig. 7a). Pearson correlation analysis revealed a negative correlation between miR-1299 and CTNND1 expression levels (Fig. 7b). We found that miR-1299 inhibition significantly increased CTNND1 protein levels and that miR-1299 mimetic agents reduced the expression of CTNND1 in HepG2 and HCCLM3 cells (Fig. 7c). The miR-1299-CTNND1 interaction was confirmed via luciferase reporter assays, where miR-1299 significantly reduced the activity of the luciferase reporter compared to the negative control, for the wildtype CTNND1 sequence. However, such reductions were not observed when the binding sites of miR-1299 were mutated (Fig. 7d). These results indicated that miR-1299 negatively regulates the expression of CTNND1.

Furthermore, miR-1299 inhibition promoted the proliferation, cell cycle progression, migration, and invasion of HCC cells. Importantly, however, these enhancements were not observed in cells co-transfected with siRNA-CTNND1 and miR-1299 inhibitor (Fig. 7e–k). These results suggested that miR-1299 targets CTNND1 and inhibits the proliferation, cell cycle progression, migration, and invasion of HCC cells.

### circMAST1 expression is positively correlated with CTNND1 expression

**Fig. 8:**
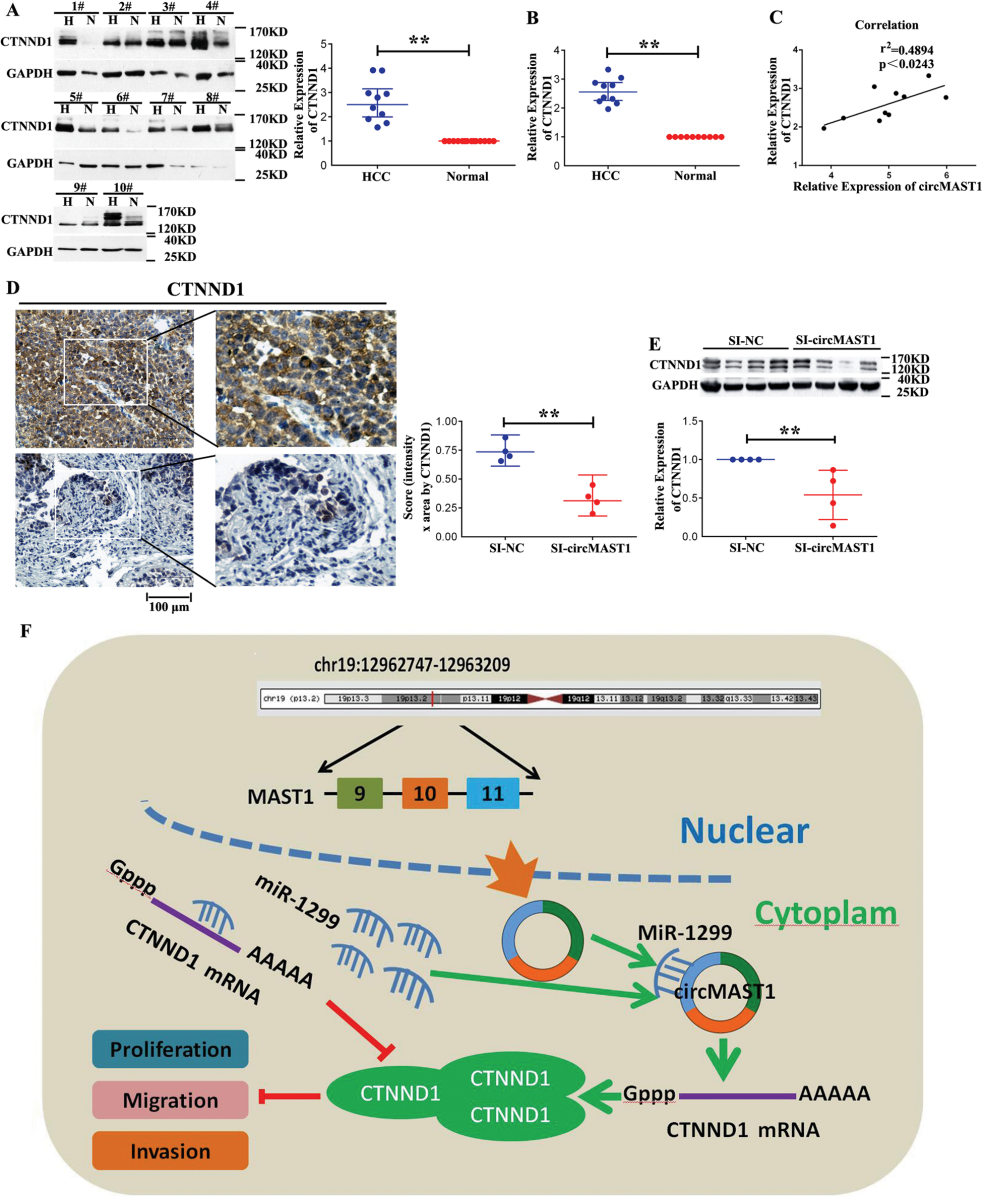
circMAST1 promotes HCC by miR-1299/CTNND1 axis. **a** The protein expression level of CTNND1 in 10 paired HCC and normal tissues (**P < 0.01; *n* = 10). **b** Relative expression of CTNND1 in HCC and normal tissue pairs were measured by qRT-PCR (**P < 0.01; *n* = 10). **c** Pearson’s correlation analyses showing the correlation of CTNND1 and circMAST1 expression (*n* = 10). **d** CTNND1 expression levels are shown in representative xenograft tumors by IHC (**P < 0.01; *n* = 4). **e** The protein expression of level of CTNND1 in xenograft model (**P < 0.01; *n* = 4). **f** Schematic diagram shows that circMAST1 promotes HCC cells proliferation、migration and invasion through miR-1299/CTNND1 axis.

To further evaluate CTNND1 expression levels, we performed qRT-PCR and western blotting in HCC and matching healthy tissues. The expression of CTNND1 was higher in HCC tissues than that of the matching healthy counterparts (Fig. 8a, b). Our aforementioned finding that the 3’ untranslated region of CTNND1 contained a miR-1299 binding site that was identical to that of circMAST1 suggested that circMAST1 might regulate CTNND1 expression by competitively binding to this miRNA. In fact, a positive correlation between circMAST1 and CTNND1 mRNA levels was found (Fig. 8c).

Immunohistochemical staining of xenografted tumors revealed that the circMAST1-silenced group showed significantly inhibited CTNND1 expression when compared to the negative control group (Fig. 8d). Western blotting results were consistent with immunohistochemical staining (Fig. 8e). These results demonstrated that circMAST1 is likely required to sustain the growth of HCC in vivo partly by regulating CTNND1. Consistent with this premise, we found that the circMAST1/miR-1299 axis also regulated CTNND1 expression, thereby influencing HCC cell proliferation, cell cycle progression, migration, and invasion (Fig. 8f).

## Discussion

Our study demonstrated that circMAST1 functions as a tumor promoter and induces HCC cancer cell proliferation and invasion through the miR-1299/CTNND1 axis, suggesting that circMAST1 is a potential biomarker and therapeutic target for HCC. This is supported by our findings: (1) circMAST1 is highly expressed in HCC tissues, and HCC cell lines (e.g., HepG2 and HCCLM3); (2) silencing circMAST1 in a murine xenograft model significantly reduces the growth of HCC; (3) circMAST1 is likely required to sustain the cell cycle progression, proliferation, migration, and invasion of HCC cell lines; (4) circMAST1 is a miR-1299 sponge, and silencing circMAST1 inhibits cell growth significantly; (5) circMAST1 sponges miR-1299 to promote CTNND1 expression and is required to sustain cancer progression. Our study thus identified a previously unrecognized role for circMAST1 in promoting HCC.

CircMAST1 (Hsa_circ_0049613) was derived from the *MAST1*. MAST1, also known as SAST170, belongs to a family of four members (MAST1–MAST4). *MAST1* rearrangement has consistently been observed in breast cancer cell lines and tissues, and overexpression of MAST1 fusion genes enhances the proliferation of breast cancer both in vitro and in vivo^28^. MAST1 was also identified as the main driver of cisplatin resistance in human cancers^29,30^; there is clinical evidence that expression of MAST1, both de novo and cisplatin-induced, contributes to platinum resistance and worse clinical outcome^30^. The aforementioned findings indicate that MAST1 plays a vital role in cancer progression. To date, however, there have been no investigations of circMAST1 and whether it plays a similar role as its parent gene in promoting the progression of cancer. In our study, we found that circMAST1 was derived from exons 9–11 of *MAST1* located on chromosome 19p13.2 and that it was dramatically upregulated in HCC cell lines and tissues relative to non-tumor tissues. Even after RNase R treatment, circMAST1 was still detected with a little degradation. Our research confirmed that circMAST1 has the same role as its parent gene in promoting HCC progression, although it is more stable.

CircMAST1 was highly expressed in HCC cell lines and tissues (most markedly in HCCLM3). More importantly, silencing circMAST1 in a murine xenograft model significantly reduced the growth of HCC. KEGG and gene ontology analyses were used to define the functional pathways of the differentially expressed circRNA host genes; these parental genes mainly participated in DNA replication and the regulation of G1/S transition. Previous studies have shown that circRNAs play an essential role in cell cycle progression and proliferation^31–34^. We provided evidence that the ectopic expression of circMAST1 is likely required to sustain cell proliferation and cell cycle progression at the G2/M phase. Concerning the specific function of circMAST1 in cell proliferation, we found that cyclin A, cyclin E, CDK1, and CDK2 expression levels were changed owing to circMAST1 silencing Our data also showed that circMAST1 silencing inhibited HCC cell migration and invasion, which are important determinants of tumor metastasis^35^. These results are consistent with those of previous studies that showed a regulatory role for circRNAs in cancer migration and invasion^36–40^.

Although the mechanisms, through which circRNA regulates carcinogenesis and cancer progression have not been fully elucidated, the “circRNA-miRNA-mRNA” axis, also known as the “miRNA sponge”, is frequently cited^41^. In our study, we confirmed that circMAST1 is a miR-1299 sponge and that silencing circMAST1 increased the expression of miR-1299 significantly, which in turn inhibited the proliferation, migration, and invasion of HCC cell lines. We also confirmed a direct correlation between miR-1299 and circMAST1. Consistent with our results, other studies have shown that circRNAs act as a sponge during the development and progression of HCC; Yu et al. also found that the circRNA cSMARCA5 sponges miR-17 and miR-181b to inhibit cancer cell proliferation and migration^17^. Many miRNAs have been shown to play a critical role in HCC initiation, development, and progression^23,42–45^. miR-1299 was shown to be significantly downregulated in HCC cells and tissues, while its overexpression inhibited cell proliferation and arrested the cell cycle in the G0/G1 phase^46^; these data were consistent with our findings. Our results indicate that circMAST1 sponges miR-1299; hence, the increased expression of circMAST1 in HCC cells leads to a decrease in the expression of miR-1299, thereby promoting proliferation, cell cycle progression, migration, and invasion. Inhibiting circMAST1 expression increased miR-1299, which consequently suppressed the proliferation, migration, and invasion of HCC cells. However, when we simultaneously inhibited the expression of circMAST1 and miR-1299, these tumorigenic properties of HCC cells were increased compared to cells in which only circMAST1 was inhibited. Our results provide evidence that miR-1299 sponging by circMAST1 appears to be a mechanism of HCC progression, and that circMAST1 is an upstream target of MiR-1299.

The role of miRNA sponging in tumor progression has previously been described^11^. We confirmed that the target gene of circMAST1-miR-1299 is *CTNND1* via bioinformatics and luciferase reporter gene analyses. A large amount of recent data has implicated CTNND1 in the regulation of cancer development and progression^35,47^, and previous studies have demonstrated that CTNND1 plays a functional role in HCC cell proliferation, migration, invasion, and metastasis. CTNND1 overexpression in HCC cells has been shown to induce epithelial-to-mesenchymal transition, migration, and invasion in vitro and also to enhance the metastatic potential in vivo^48,49^. While our data are consistent with those of these previous studies, we additionally revealed that circMAST1-miR-1299 regulates CTNND1 as part of a sponging mechanism. Inhibition of miR-1299 expression promoted CTNND1 expression, which in turn increased the proliferation, migration, and invasion of HCC cells. The simultaneous inhibition of miR-1299 and CTNND1 also caused an attenuation in the tumorigenic features of HCC cells to a greater extent when compared to miR-1299 inhibition alone. To our knowledge, our study is the first to show that circMAST1 is involved in the expression of CTNDD1. Moreover, we demonstrated that circMAST1 silencing in nude mice significantly reduced the expression of CTNND1. These findings suggest that circMAST1 protects CTNND1 from miR-1299-mediated degradation in a competing endogenous RNA-mediated fashion.

We acknowledge that our research still has limitations. The research clarifies circMAST1 function as a sponge of miR-1299 to promote CTNND1-induced HCC cancer cell proliferation and invasion. However, circRNAs may have other mechanisms by which they might regulate the development and progression of HCC. For example, circRNAs have been shown to regulate parental gene expression, and the expression of peptides/proteins in other cancers^14,40^; the role of circMAST1 remains to be explored in HCC. In terms of clinical diagnosis and treatment, we still need to perform more experimental work, including expanding the sample size and expression stability of circMAST1 in the peripheral blood of patients with HCC and evaluating the beginning of the high expression among the stages of HCC.

## Conclusions

Our study demonstrated that circMAST1 is upregulated in HCC cell lines and tissues and that its high expression is associated with HCC progression. CircMAST1 is required to sustain the proliferation and invasion of HCC by directly binding to miR-1299 and impeding its suppression of CTNND1. Our findings suggest that circMAST1 is potentially a novel biomarker and therapeutic target for HCC.

## Methods

All supporting data are available within the article and its online-only Supplement Data, which also includes Expanded Methods.

### Patient selection

A total of 39 HCC samples were obtained from the clinical sample bank of the First Affiliated Hospital, Harbin medical university. The collection of human specimens was approved by the Biomedical Ethics Committee of the Harbin medical university. First Affiliated Hospital and written informed consent was obtained from each patient. Inclusion criteria for patient selection were curative hepatectomy performed between 2017 and 2018. This study was performed in compliance with the Declaration of Helsinki and was approved by the Institutional Review Board of the Harbin Medical University.

### Animal studies

All protocols were approved by the Harbin Medical University Animal Care and Use Committee. This study was also approved by the ethics review board of Harbin Medical University.

### Statistical analyses

All statistical analyses were performed using SPSS version 21.0 (IBM SPSS Inc., Chicago, IL, USA) and GraphPad Prism version 6.0 (GraphPad Software, LaJolla, CA, USA) software. Categorical variables are expressed as a count or percentage and tested using chi-square or Fisher’s exact tests, as appropriate. Continuous data are reported as mean ± standard deviation (SD) and compared using Student’s t-test, the one-way analysis of variance (ANOVA) test or Mann-Whitney test as appropriate. Correlations were calculated using Pearson’s correlation analysis. *P* < 0.05 was considered statistically significant.

## Supplementary information

Supplement Materials and Methods

Supplement Materials and Methods-Additional file 2 Table S1. Clinical Characteristics of 39 HCC Patients

Supplement Materials and Methods-Additional file 3 Table S2. Sequence of primers for qRT-PCR

Supplement Materials and Methods-Additional file 4 Table S3. Antibody for western blotting and immunohistochemistry

Supplement Materials and Methods-Additional file 5 Table S4. Target sequences of siRNA

Supplemental Figure 1

Supplemental Figure 2

Supplementary Figure Legends
