# Supplementary material for: The circular RNA circMAST1 promotes hepatocellular carcinoma cell proliferation and migration by sponging miR-1299 and regulating CTNND1 expression
Source: Cell Death Dis. 2020 May 11;11(5):340. doi: 10.1038/s41419-020-2532-y (PMC7214424; doi:10.1038/s41419-020-2532-y)
Supplement: Supplementary file 1 — Supplement Materials and Methods [file 41419_2020_2532_MOESM1_ESM.docx]

**Online data supplement**

**Additional file 1: Supplementary Materials and Methods**

***Gene Expression Omnibus (GEO) dataset***

We downloaded the expression microarray data (CEL data) from the GSE78520 dataset of the GEO (http://ncbi.nlm.nih.gov/geo/).

**Cell lines and clinical tissues**

A total of 39 HCC samples were obtained from the clinical sample bank of the First Affiliated Hospital, Harbin medical university. The collection of human specimens was approved by the Biomedical Ethics Committee of the Harbin medical university. First Affiliated Hospital and written informed consent was obtained from each patient. Inclusion criteria for patient selection were curative hepatectomy performed between 2017 and 2018.All the patients were pathologically diagnosed with hepatocellular carcinoma. Their liver specimens were evaluated by pathologists and their clinical stages were determined, according to the TNM classiﬁcation. HCC patients with the following conditions were excluded: (1) patients≤18 or≥70 years of age or without full civil capacity; (2) patients with a history of preoperative anticancer radiotherapy or chemotherapy, biological, immune, and traditional Chinese medicine; (3) patients with incomplete postoperative follow-up data; (4) patients with a history of another organ malignancy, or systemic immune disease. All specimens were collected within 15 min after removal from the body and were immediately snap-frozen in liquid nitro- gen before storage at −80 °C. 15 pairs were used to compare the expression levels of the genes of interest between HCC and paired non-tumorous tissues.

All cell lines used in this study (HepG2, Huh7, HCCLM3, SK-Hep1, LO2) were purchased from the Cell Bank of Type Culture Collection (Chinese Academy of Sciences, Shanghai, China). All cells were cultured in DMEM/high glucose medium (Hyclone, Logan, UT, USA) supplemented with 10% foetal bovine serum (PAN-Biotek, Aidenbach, Bavaria) and 1% penicillin-streptomycin (Hyclone) in a humidified atmosphere at 37 °C containing 5% CO2.

**Xenograft nude mouse model**

Six-week-old male BALB/C nude mice purchased from Weitonglihua (Beijing, China) were maintained under specific pathogen-free conditions with a 12-h light/dark cycle. All animal experiments were performed in accordance with a statement of compliance with ethical regulations and approved by the Biomedical Ethics Committee of the Harbin medical university.Animals are grouped randomly during the experiment. HCCLM3 cells were subcutaneously injected into the right upper back of the nude mice (1×10^6^ cells per mouse) for 12 days, after subcutaneous incubation of HCC tumor mass.Then, 10 nmol cholesterol-modified circMAST1 siRNA or control siRNA RiboBio (Guangzhou, China) were intratumorally injected every 3 days for 24 days. 36 days later, the mice were sacrificed and tumour tissues were collected for examination of the parameters of interest.

*Circular structure confirmation*

The circular structure of circMAST1 was confirmed by Sanger sequencing, divergent primer PCR and RNase R treatment. PCR products, amplified by divergent primers of circMAST1, were inserted into the T vector and delivered to SinoGENE for Sanger sequencing. The results were crosschecked with the back spliced region of circMAST1 supplied by circBASE [^1^](#_ENREF_1). For RNase R treatment, 3 μg total RNA extracted from HCC was incubated with 10 U RNase R (20 U/μl, Epicentre, Madison, WI, USA) in a 10 μl total volume at 37 °C for 45 min, followed by an incubation at 70 °C for 10 min to deactivate the RNase R. The treated RNAs were used for qRT-PCR [^2^](#_ENREF_2).

**Quantitative real-time polymerase chain reaction analysis for serum**

The total serum RNA was extracted from 200ul HCC and normal control serum by using GenEluteTM Plasma/ Serum RNA Purification Mini Kit (Cat. RNB500) (SigmaAldrich, MO). and complement DNA was generated using the and cDNA was generated using the First Strand cDNA Synthesis kit ReverTra Ace (TOYOBO, JAPAN) after RNA quantification. The qRT-PCR assays were performed using Power SYBB Green PCR Master Mix((Life Technologies, Carlsbad, CA, USA), The CircRNA and gene expression levels were normalized to that of GADPH. The relative expression was analyzed by the comparative cycle threshold (Ct) method, according to the equation 2-Δ^Ct^ [Δ^Ct^ = Ct-Ct (GAPDH)]. All experiments were performed in triplicate.

**Quantitative real-time polymerase chain reaction analysis, western blotting analysis, and immunofluorescence assays**

Total RNA was extracted from HCC cell lines and tissue using Trizol solution, and complement DNA was generated using the Golden 1st cDNA Synthesis kit (Haigene, China) after RNA quantification. The qRT-PCR assays were performed using Power SYBB Green PCR Master Mix((Life Technologies, Carlsbad, CA, USA), The CircRNA and gene expression levels were normalized to that of GADPH. The miRNA expression levels were normalized to that of U6. Each sample was tested in triplicate. The relative expression was analyzed by the comparative cycle threshold (Ct) method, according to the equation 2-Δ^Ct^ [Δ^Ct^ = Ct-Ct (GAPDH)].The primer sequences of circ_0000026, circ_0001410, circ_0000520, circ_0077248, circ_0072088, circ_0003028, circ_0007928, circ_0004891, circ_0007646, mir1299,U6 and GADPH was designed by RiboBio (Guangzhou, China). The CTNND1 and MAST1 was designed by Genscript (Nanjing, China).All experiments were performed in triplicate. The primers used in this study are listed in Additional file 3: Table S2.

For western blotting, the total protein extracts from cells were separated by sodium dodecyl sulfate-polyacrylamide gel electrophoresis (SDS-PAGE), transferred onto polyvinylidene difluoride membranes, and incubated with the corresponding antibodies. The membranes were developed using the enhanced chemiluminescence method (Haigene, China). The antibodies used in this study are listed in Additional file 4: Table S3.

Fresh samples were cut to an appropriate size and fixed in 4% paraformaldehyde for 24 h. The fixed specimens were dehydrated in a graded series of ethanol solutions, embedded in paraffin and cut at a thickness of 4 μm. The sections were dewaxed and rehydrated using xylene and ethanol, and high-pressure heat was applied for antigen retrieval. The sections were incubated with the first antibody overnight at 4 °C. Finally, all sections were dehydrated, cleared, mounted, and visualised with a diaminobenzidine-based colorimetric method. The antibodies used in this study are listed in Additional file 4: Table S3.

*Fluorescence in situ hybridization (FISH)*

In situ hybridization was performed with a FISH Kit (RiboBio, Guangzhou, China). Cells, frozen sections of HCC, and paired adjacent liver tissues were brieﬂy rinsed in PBS and fixed in 4% formaldehyde for 10 min. Then the cells were permeabilized in PBS containing 0.5% Triton X 100 at 4 °C for 5 min, washed with PBS three times for 5 min, and prehybridized at 37 °C for 30 min before hybridization. Then an anti-Circ_0049613, anti-U6, or anti-18S oligodeoxynucleotide probe (RiboBio, Guangzhou, China) was used in the hybridization solution at 37 °C overnight in the dark. The next day, the cells were counterstained with DAPI and imaged using a NA1.4 inverted Leica DMI6000 microscope (Leica, Heidelberg, Germany), and images were visualized using a Hamamatsu ORCA-R2 camera (Hamamatsu, Japan) and recorded using LAS AF software (Leica). The experiments were conducted in triplicate.

*Cell cycle and DNA analysis*

The Cycle TEST PLUS DNA Reagent Kit was used to examine whether circMAST1 influenced the cell cycle. We analyzed the proportion of cells in the G0/G1, S and G2/M phases by flow cytometry as reported recently. The cell cycle and DNA analysis procedures were described previously [^3^](#_ENREF_3).

**Cell Proliferation, migration, and Matrigel invasion assay**

Cell proliferation was assessed using the WST-1 assay (Beyotime Biotechnology, Nantong, China). Cells (2×10^3^) were seeded into each well of 96-well plates. 10 μl of WST-1 solution was added to each well at six time points. After 4 h of incubation at 37 °C, the absorbance at 450 nM was measured using Spectra Max 250 spectrophotometer (Molecular Devices, Sunnyvale, CA, USA). Experiments were independently performed in triplicate.

For the colony formation assays, cells (1×10^2^) were suspended and plated into each well of 6-well plates. After 14 days incubation at 37 °C in a chamber with an atmosphere of 5% CO2, colonies were fixed with 1ml of 4% paraformaldehyde (Solarbio, Beijing, China) for 30 min and were stained with crystal violet (Beyotime Biotech-nology, Nantong, China) for 25 min. Colonies were counted after photographing the sample (Nikon, Tokyo, Japan).

Cell migration and invasion were measured using a transwell migration assay and a Matrigel invasion assay. For the transwell migration assay, 2–4×10^5^ cells were suspended in 200 µl of DMEM without serum and placed in the cell culture insert (8 µm pore size; BD Falcon, San Jose, CA) of a companion plate (BD Falcon) with a prewarmed culture medium containing 20% fetal bovine serum in the well. The cells were incubated for 24h at 37°C in 5% CO2 and were then fixed with 4% paraformaldehyde in PBS.

For the Matrigel invasion assay, 2–4×10^5^ cells were suspended in 200 µl of DMEM without serum and were placed in the cell culture insert precoated with 50µl Matrigel ((BD Biosciences, San Jose, CA, USA). A prewarmed culture medium containing 20% fetal bovine serum was added to the well. The cells were incubated for 48h at 37°C in 5% CO2 and were then fixed with 4% paraformaldehyde in PBS. The nonmigrated or invaded cells on the top of the membrane were gently removed with a cotton swab. Cell migration or invasion was determined by staining cells with 0.1% crystal violet (Sigma, St Louis, MO) and counting the cells under a light microscope (200x magnification) in eight randomly selected areas.

**Transfection experiment**

siRNA of circMAST1 was synthesized by RiboBio (Guangzhou, China). inhibitor of he miR-1299 mimics and negative control, miRNA_1299, siRNA of CTNND1 was synthesized by Gene Pharma (Shanghai, China). HepG2 and HCCLM3 cells were transfected with siRNA of circMAST1 using the Lipofectamine 2000® siRNA transfection reagent following the manufacturer’s protocol. The target sequences of siRNAs are listed in Additional file 5: Table S4.

***Dual luciferase assay***

Targeted binding of circ_0049613 to miR-1299 was predicted using bioinformatics websites, including RNAhybrid, regRNA, and miRanda. Targeted binding of miR-1299 to catenin delta-1 (CTNND1) was predicted using bioinformatics websites, including TargetScan, miRDB, miRWALK and miRanda. The full-length sequences of circ_0049613 with and without mutated predicted miR-1299 binding site was subcloned into pmirGLO reporter vector (Promega, WI, USA). The full-length sequences of CTNND1 with and without mutated predicted miR-1299 binding site were subcloned into pmirGLO reporter vector (Promega, WI, USA). Then, the Lipo2000 was used for transfection of the sequences into 293T cells. Finally, luciferase activity was measured using the dual luciferase assay kit.

**References**

1 Glazar, P., Papavasileiou, P. & Rajewsky, N. circBase: a database for circular RNAs. *RNA* **20**, 1666-1670, (2014).

2 Xu, L. *et al.* CircSETD3 (Hsa_circ_0000567) acts as a sponge for microRNA-421 inhibiting hepatocellular carcinoma growth. *J Exp Clin Cancer Res* **38**, 98 (2019).

3 Yu, X. *et al.* Growth Differentiation Factor 11 Promotes Abnormal Proliferation and Angiogenesis of Pulmonary Artery Endothelial Cells. *Hypertension* (2018).
