# Supplementary material for: The circular RNA circMAST1 promotes hepatocellular carcinoma cell proliferation and migration by sponging miR-1299 and regulating CTNND1 expression
Source: Cell Death Dis. 2020 May 11;11(5):340. doi: 10.1038/s41419-020-2532-y (PMC7214424; doi:10.1038/s41419-020-2532-y)
Supplement: Supplementary file 2 — Supplement Materials and Methods-Additional file 2 Table S1. Clinical Characteristics of 39 HCC Patients [file 41419_2020_2532_MOESM2_ESM.docx]

**Additional file 2: Table S1. Clinical Characteristics of 39 HCC Patients According to circMAST1 Expression Level**

| **Variable** | **circMAST1**  **High Low** | **P-value** |
| --- | --- | --- |
| All cases | 20 19 |  |
| Age, years,>50: ≤50 | 12:6 13:4 | 0.7112 |
| Gender, male/female | 19:1 16:3 | 0.3416 |
| HBsAg, positive/negative | 12:8 13:6 | 0.7411 |
| AFP, µg/L,>400: ≤400 | 5:15 6:13 | 0.7311 |
| Tumour size, cm, >3: ≤3 | 18:2 17:2 | 1 |
| Microvascular invasion, yes:no | 15:5 18:1 | 0.1818 |
| Lymphonodemetastasis, yes:no | 0:20 2:17 | 0.2308 |
| TNM stage, I ~ II: III ~Ⅳ | 11:9 13:6 | 0.5145 |

χ^2^ test was used to test the association between two categorical variables.

* Statistically significant.
