# Supplementary material for: The circular RNA circMAST1 promotes hepatocellular carcinoma cell proliferation and migration by sponging miR-1299 and regulating CTNND1 expression
Source: Cell Death Dis. 2020 May 11;11(5):340. doi: 10.1038/s41419-020-2532-y (PMC7214424; doi:10.1038/s41419-020-2532-y)
Supplement: Supplementary file 3 — Supplement Materials and Methods-Additional file 3 Table S2. Sequence of primers for qRT-PCR [file 41419_2020_2532_MOESM3_ESM.docx]

**Additional file 3: Table S2. Sequence of primers for qRT-PCR.**

| **Gene** | **Forward primer (5’-3’)** | **Reverse primer(5’-3’)** |
| --- | --- | --- |
| h-hsa_circ_0000026 | CCCCTGGACTCTCTCAACAAT | TGAGACAGCGGGAGTGAAGAT |
| h-hsa_circ_0001410 | AGGTGCTTGGAATGGTTCTATG | GCCAATGTCTTCTGTTAGGTTCA |
| h-hsa_circ_0000520 | TGTCCCTTGGGAAGGTCTGA | GGAGTGACAGGACGCACTCA |
| h-hsa_circ_0077248 | TGCTACTTGAAAACTACCAGCCA | GCACAGCTATGACCTTGAGGAA |
| h-hsa_circ_0072088 | TGCTCTACGCCACGCTAAGT | ACGACGTAAGGGCCAGCTT |
| h-hsa_circ_0049613 | CCCTGAGCAAGACGATCTCTC | AGGGCGTGAGATGATAATAAGC |
| h-hsa_circ_0003028 | CGAATCTCTCCGCATGTAGAGC | CAAAGAGATCCTCCTGGTGATATG |
| h-hsa_circ_0007928 | GTTGAACCAGAAAACATTTTCAGC | CCGCAGACTTCCTGTTTGG |
| h-hsa_circ_0004891 | GTTTGAGAGTGGGAACATGACG | TGGAGCGGTTGATCTTGGG |
| h-hsa_circ_0007646 | GTTATGCTTGTCCTAGCTTGGAA | TTGCCAGTGTTGAGAGATGAGA |
| h-hsa_circ_0005075 | CCTGGACTCTCTCAAAATTCCTAG | CCACTTGTGTTACCGTGCTGT |
| MAST1 | CTCTGGACCGCGCTTTCTAAT | AGCTGAGTAACGAAGGCCAC |
| CTNND1 | CCAAGAAGGGCAAAGGGAAA | AGGCTTCTAGGATGGCAGGA |
| GAPDH | CACCCACTCCTCCACCTTTG | CCACCACCCTGTTGCTGTAG |
| U6 | CGCTTCGGCAGCACATATAC | TTCACGAATTTGCGTGTCATC |
| hsa-miR-1299 | CCCTAACGGTTCTGGAATTCTGT | TATGGTTGTTCACGACTCCTTCAC |

**Abbreviations:** qRT-PCR, quantitative real-time polymerase chain reaction.
