# Supplementary material for: The circular RNA circMAST1 promotes hepatocellular carcinoma cell proliferation and migration by sponging miR-1299 and regulating CTNND1 expression
Source: Cell Death Dis. 2020 May 11;11(5):340. doi: 10.1038/s41419-020-2532-y (PMC7214424; doi:10.1038/s41419-020-2532-y)
Supplement: Supplementary file 4 — Supplement Materials and Methods-Additional file 4 Table S3. Antibody for western blotting and immunohistochemistry [file 41419_2020_2532_MOESM4_ESM.docx]

**Additional file 4: Table S3. Antibody for western blotting and immunohistochemistry.**

| **Antibody Dilution** | **Company** | **Cat No.** |
| --- | --- | --- |
| PCNA 1:250 | BOSTER | BM0104 |
| cyclin A 1:250 | BOSTER | A00700 |
| cyclin E 1:400 | BOSTER | A00543-1 |
| Cyclin-dependent kinases1(CDK1) 1:400 | BOSTER | BM0027 |
| Cyclin-dependent kinases2(CDK2) 1:400 | BOSTER | PB0562 |
| CTNND1 1:400 | BOSTER | P02333 |
| GAPDH 1:1000 | BOSTER | BM3876 |
| PECAM(CD31) 1:100 | santa | BA2966 |
| Ki-67 1:200 | BOSTER | PB0065 |
| HRP-labeled Goat Anti-Rabbit IgG(H+L) | Beyotime | A0208 |
| HRP-labeled Goat Anti-mouse IgG(H+L) | Beyotime | A0216 |
