# Supplementary material for: The circular RNA circMAST1 promotes hepatocellular carcinoma cell proliferation and migration by sponging miR-1299 and regulating CTNND1 expression
Source: Cell Death Dis. 2020 May 11;11(5):340. doi: 10.1038/s41419-020-2532-y (PMC7214424; doi:10.1038/s41419-020-2532-y)
Supplement: Supplementary file 5 — Supplement Materials and Methods-Additional file 5 Table S4. Target sequences of siRNA [file 41419_2020_2532_MOESM5_ESM.docx]

**Additional file 5: Table S4. Target sequences of siRNA.**

| **siRNA** | **Target sequence** |
| --- | --- |
| si-hsa_circ_0049613(MAST1)_001 | CGATCTCTCTGAGGCCTAT |
| si-hsa_circ_0049613(MAST1)_002 | TCTCTCTGAGGCCTATGAA |
| si-hsa_circ_0049613(MAST1)_003 | TGAGGCCTATGAACGCTCT |
| MircoRNA inhibitor N.C. | CAGUACUUUUGUGUAGUACAA |
| Hsa-miR-1299 inhibitor | UCCCUCACACAGAAUUCCAGAA |
| Hsa-CTNND1- 1882 | GACGUGACCAGGAUAACAATT |
|  | UUGUUAUCCUGGUCACGUCTT |
| Hsa-CTNND1-1446 | GACCUGGAUUAUGGUAUGATT |
|  | UCAUACCAUAAUCCUGGUCTT |
| Hsa-CTNND1-1105 | GUCGUGAUUUCCUCAAGAATT |
|  | UUCUUGCGGAAAUCACGACTT |
| NC | UUCUCCGAACGUGUCACGUTT |
|  | ACGUGACACGUUCGGAGAATT |
| Hsa-miR-1299 mimics | UUCUGGAAUUCUGUGUGAGGGA |
|  | CCUCACACAGAAUUCCAGAAUU |
