# Supplementary figures and images for: The circular RNA circMAST1 promotes hepatocellular carcinoma cell proliferation and migration by sponging miR-1299 and regulating CTNND1 expression
Source: Cell Death Dis. 2020 May 11;11(5):340. doi: 10.1038/s41419-020-2532-y (PMC7214424; doi:10.1038/s41419-020-2532-y)

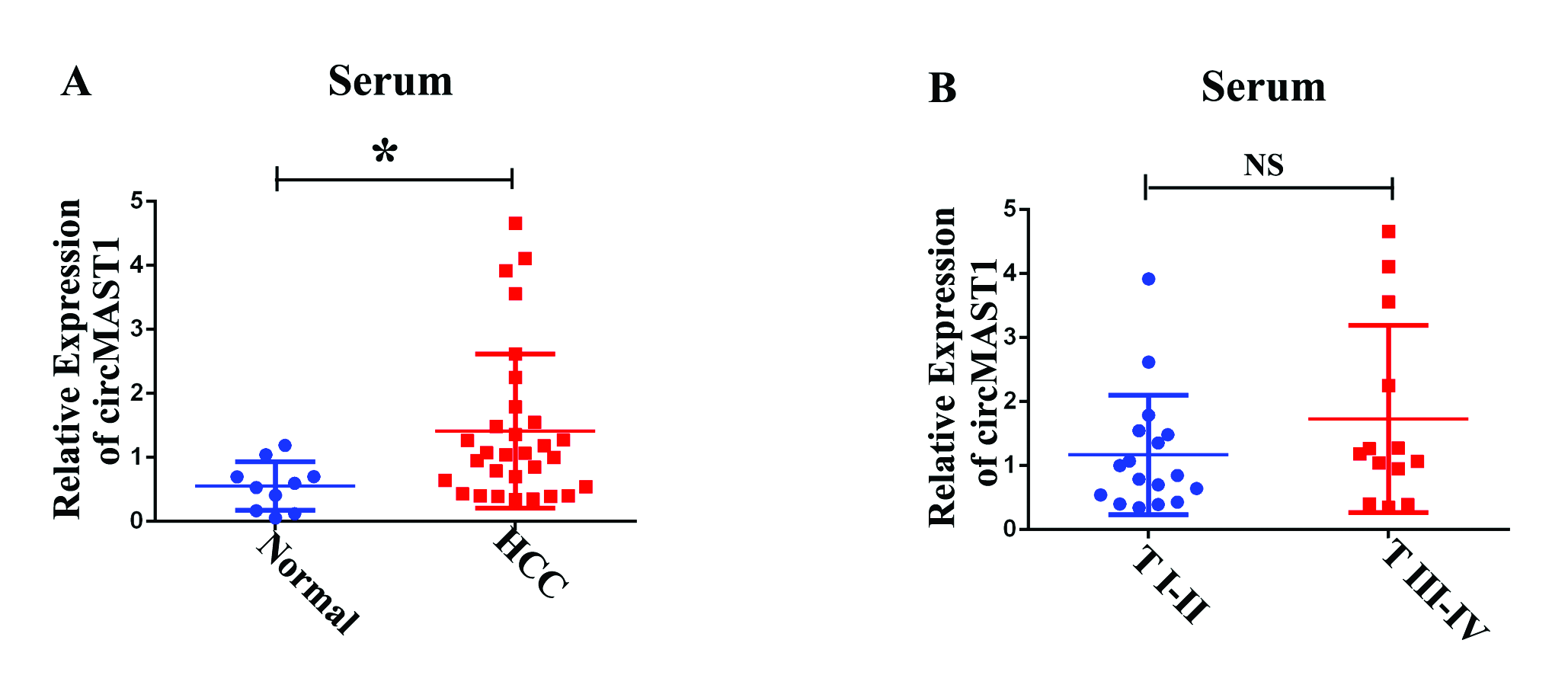

Supplement: Supplementary file 6 — Supplemental Figure 1 [file 41419_2020_2532_MOESM6_ESM.tif]

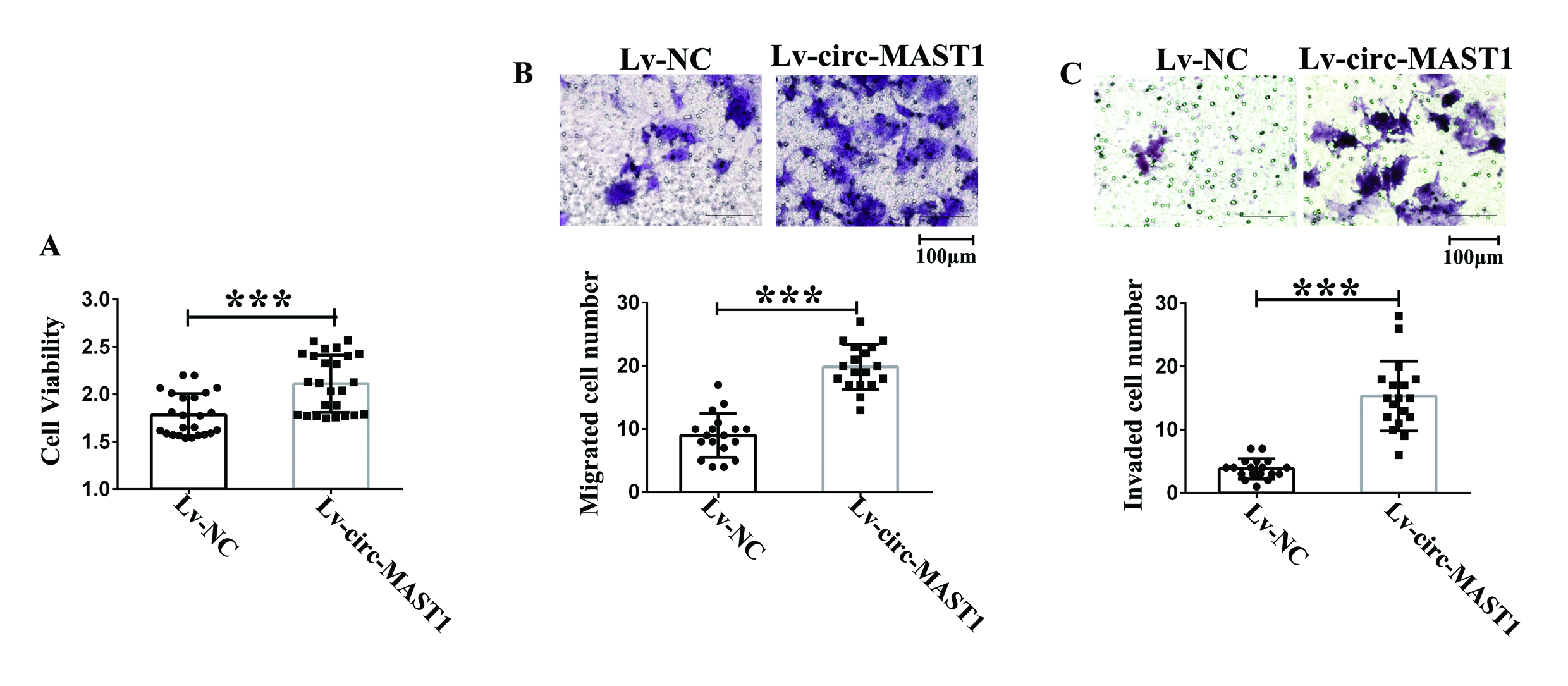

Supplement: Supplementary file 7 — Supplemental Figure 2 [file 41419_2020_2532_MOESM7_ESM.tif]
