# Supplementary material for: The circular RNA circMAST1 promotes hepatocellular carcinoma cell proliferation and migration by sponging miR-1299 and regulating CTNND1 expression
Source: Cell Death Dis. 2020 May 11;11(5):340. doi: 10.1038/s41419-020-2532-y (PMC7214424; doi:10.1038/s41419-020-2532-y)
Supplement: Supplementary file 8 — Supplementary Figure Legends [file 41419_2020_2532_MOESM8_ESM.docx]

**Supplementary Figures:**

**Supplemental Figure 1. A：**The existence of circMAST1 in HCC and health control serum was detected by using real-time PCR (**P*<0.05; n=10). **B:** The comparison between the expression level of circMAST1 in patients’ serum.

**Supplemental Figure 2. A:** Cell proliferation ability of Huh7 cells transfected with Lv-circMAST1 or Lv-NC was evaluated by WST-1 assay (****P*<0.001; n=3). **B：** Cell migration capability of Huh7 cells transfected with Lv-circMAST1 or Lv-NC was assessed by transwell migration (****P*<0.001; n=3). **C:** Cell invasion capability of cells transfected with Lv-circMAST1 or Lv-NC was evaluated by matrigel nivasion assays(****P*<0.001; n=3).
